# C-arm cone-beam CT parenchymal blood volume imaging for transarterial chemoembolization of hepatocellular carcinoma: implications for treatment planning and response

**DOI:** 10.1186/s41747-019-0099-0

**Published:** 2019-05-29

**Authors:** Rory L. O’Donohoe, Richard G. Kavanagh, Alexis M. Cahalane, Diarmaid D. Houlihan, Jeffrey W. McCann, Edmund Ronan Ryan

**Affiliations:** 1 Department of Radiology, St Vincent’s University Hospital, Elm Park, Dublin 4, Ireland; 2 Department of Hepatology, St Vincent’s University Hospital, Elm Park, Dublin 4, Ireland

**Keywords:** Blood volume, Carcinoma (hepatocellular), Chemoembolization (therapeutic), Cone-beam computed tomography, Multidetector computed tomography

## Abstract

We report on the feasibility of C-arm cone-beam computed tomography (CBCT) parenchymal blood volume imaging (PBVI) performed immediately following transarterial chemoembolization (TACE) of hepatocellular carcinoma (HCC) to assess the need for repeat treatment. Eighteen TACE procedures were included. A retrospective assessment was made for the presence or absence of residual disease requiring treatment on immediate post-TACE PBVI and on interval follow-up multidetector computed tomography (MDCT) or magnetic resonance imaging (MRI). In 9/18 cases, both PBVI and MDCT/MRI showed that no further treatment was required. In 6/18 cases, further treatment was required on both PBVI and MDCT/MRI. In three cases, PBVI showed that further treatment was not required but MDCT/MRI showed residual disease requiring repeat treatment. There were no cases with PBVI showing residual disease not detected on follow-up MDCT/MRI. The PBVI sensitivity for detecting disease requiring repeat TACE was 67% (95% confidence interval [CI] 30–93%), and specificity was 100% (95% CI 66–100%). The use of C-arm CBCT PBVI for the detection of residual viable tumor within a treated lesion immediately after TACE is feasible. It may allow repeat TACE to be planned without performing interval imaging with MDCT or MRI.

## Key points

- C-arm cone-beam computed tomography (CBCT) parenchymal blood volume imaging (PBVI) can be performed immediately after transarterial chemoembolization (TACE) to assess residual hepatocellular carcinoma (HCC) perfusion, when the patient is still in the angiography suite.
- The presence of residual HCC perfusion may allow repeat TACE to be planned without interval cross-sectional imaging with multidetector computed tomography (MDCT) or magnetic resonance imaging (MRI).
- The absence of residual HCC perfusion should be confirmed with follow-up MDCT or MRI due to the possibility of false negatives on C-arm CBCT PBVI.

## Background

Hepatocellular carcinoma (HCC) is the most common primary malignancy of the liver and has a rising incidence. It is the fifth most common malignancy worldwide and the third most common cause of cancer-related death [1]. Transarterial chemoembolization (TACE) is a widely accepted therapy in unresectable HCC and provides a proven survival benefit [2, 3]. The Barcelona Clinic Liver Cancer (BCLC) staging system considers TACE to be the standard of care in intermediate stage (BCLC stage B) disease [4], and TACE is also frequently employed as a bridge to liver transplant. Assessment of response to treatment is typically performed using follow-up multi-phase multidetector computed tomography (MDCT) or magnetic resonance imaging (MRI).

C-arm cone-beam computed tomography (CBCT) can be performed prior to TACE to facilitate lesion localization and procedure planning, and has been demonstrated to provide additional information to digital subtraction angiography (DSA) impacting treatment in more than a quarter of cases [5]. TACE performed with C-arm CBCT has been shown to improve survival when compared with TACE with DSA alone [6]. In addition to the usefulness of C-arm CBCT prior to TACE, it has shown promise for the immediate assessment of tumor response following treatment [7].

A novel C-arm CBCT post-processing technique allowing intra-procedural color-map depiction and quantitative measurement of parenchymal blood volume in HCC before and after chemoembolization has been introduced. Initial data suggested that C-arm CBCT parenchymal blood volume imaging (PBVI) might have a role in the immediate post-TACE assessment of treatment response [8, 9].

The purpose of this study is to report on the feasibility of C-arm CBCT PBVI immediately following TACE of HCC to assess the need for repeat treatment.

## Methods

### Patients

Ethical approval for this retrospective study was obtained from the institutional ethics and medical research committee. Informed consent was obtained in all cases. A total of 43 TACE procedures utilizing C-arm CBCT PBVI were performed on 25 patients between June 17, 2014 and August 7, 2015. Of these, 18 TACEs on 13 patients were evaluated with both immediate post-TACE PBVI and a subsequent multi-phase MDCT or MRI for assessment of treatment response, without intervening TACE. All 18 TACE procedures were included. Of the 13 patients, 12 were male and one was female. Patient age ranged from 31 to 73 years (median 62). All patients were BCLC stage A or B at the time of treatment. The underlying diagnoses were alcohol-related liver disease (5 patients), hepatitis B infection (3 patients), hepatitis C infection (3 patients), hemochromatosis (1 patient) and alpha 1-antitrypsin deficiency (1 patient).

### TACE protocol

In all cases, prior MDCT angiography or MRI angiography was evaluated to identify variant hepatic arterial anatomy and assess for possible extra-hepatic arterial tumor supply. All procedures were performed by one of two operators (J.McC. or E.R.R.), with procedural details depending on operator preference. A 5-Fr or 6-Fr right common femoral artery sheath was placed with ultrasound guidance. A 5-Fr hydrophilic C2 catheter (Terumo, Leuven, Belgium) or Sim1 catheter (Boston Scientific, Marlborough MA, USA) was used to cannulate the superior mesenteric artery; DSA was performed with delayed images to confirm portal vein patency. The coeliac artery was then cannulated and the hydrophilic C2 catheter was advanced into the proper hepatic artery or, in cases using a Sim1, a microcatheter (Renegade, Boston Scientific, Marlborough MA, USA) was advanced into the proper hepatic artery. C-arm CBCT PBVI was then performed through the C2 catheter or microcatheter as outlined later. Super-selective drug-eluting bead TACE (DEB-TACE) was performed in each case by using the microcatheter system to access the hepatic arterial branch supplying the targeted lesion or lesions. Fourteen TACEs were performed using doxorubicin loaded on 100–300-μm DC-Beads (BTG, Farnham, United Kingdom); in one of these 14 cases, the treatment was supplemented with bland embolization using 500–700-μm Embosphere particles (Merit Medical, Paris, France). The supplementation with bland embolic was in a case where residual tumor enhancement was visible on DSA despite the entirety of the available chemo-embolic having been administered. Three treatments were performed using doxorubicin loaded onto 50–100-μm Hepasphere beads (Merit Medical, Paris, France). One treatment used doxorubicin loaded on 30–60-μm Hepasphere beads (Merit Medical, Paris France). Chemoembolization was performed until slow forward flow in all cases.

### C-arm CBCT and PBVI protocol

**Fig. 1:**
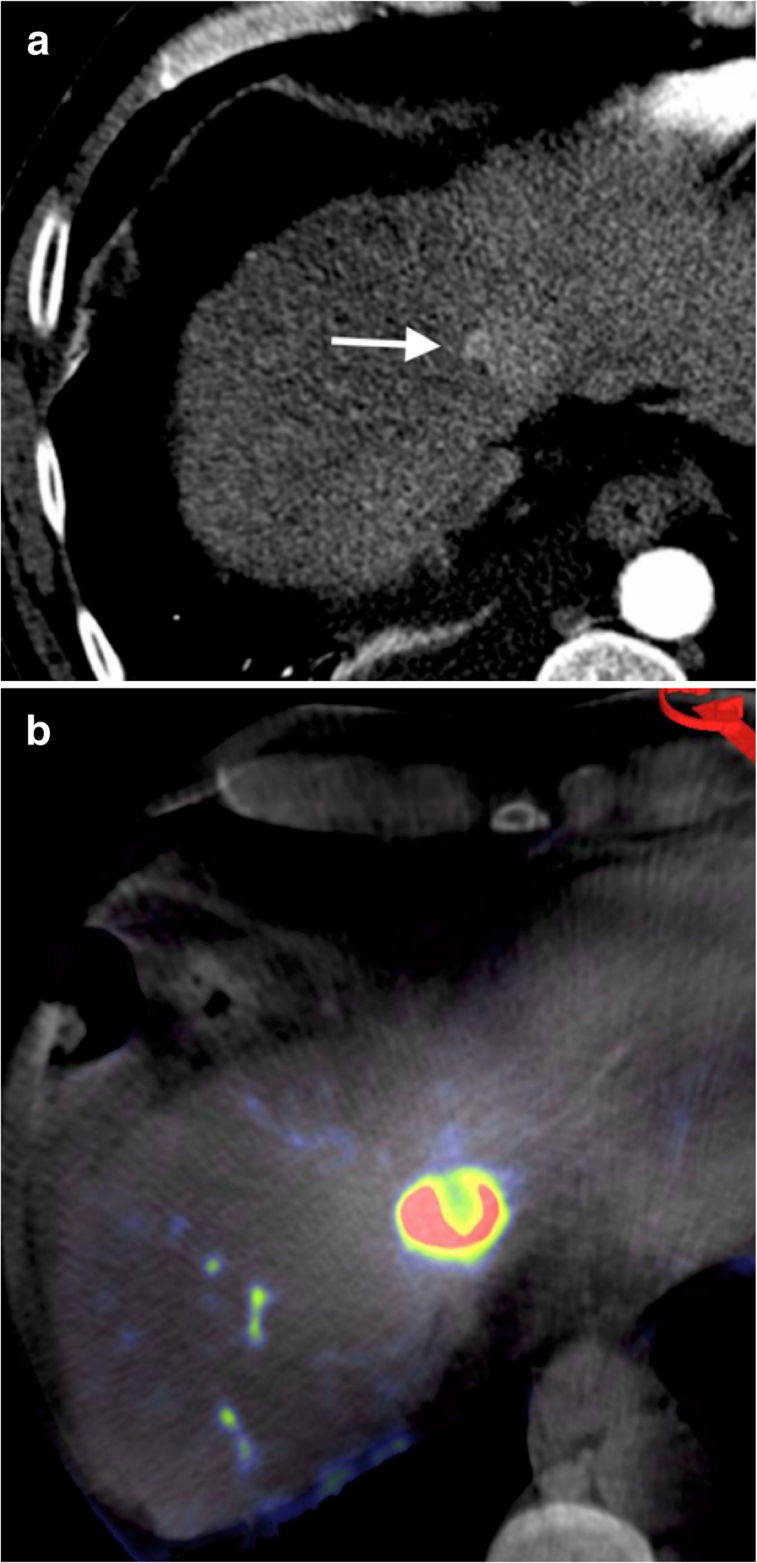
**a** Pre-TACE arterial phase MDCT showing HCC in segment IVa/VIII (arrow). **b** Pre-TACE C-arm CBCT PBVI showing the same lesion

**Fig. 2:**
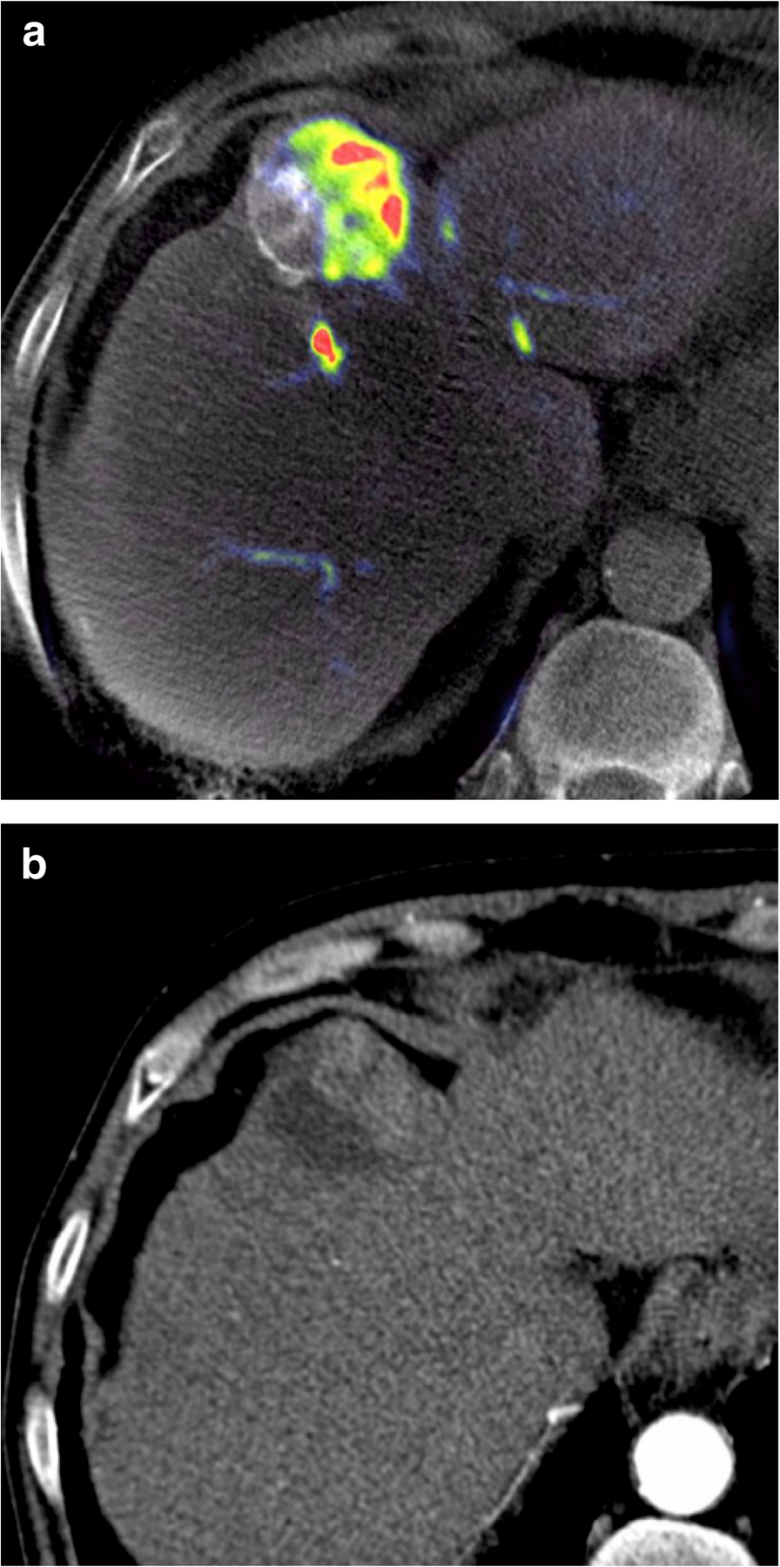
**a** C-arm CBCT PBVI performed immediately after TACE via the artery to segment VIII showing high density contrast in the treated portion of the lesion. There is persistent viable tumor supplied via segment IVa. **b** Follow-up arterial phase MDCT confirming persistent viable tumor supplied via segment IVa

**Fig. 3:**
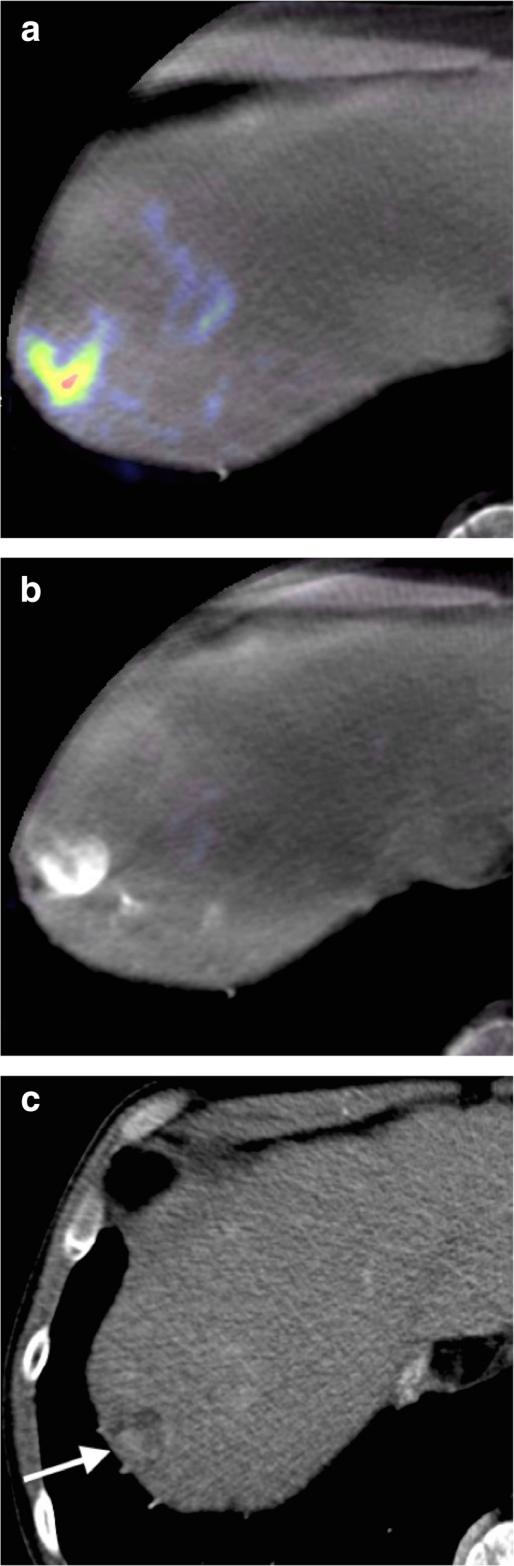
**a** Pre-TACE C-arm CBCT PBVI via the proper hepatic artery showing viable tumor. TACE has previously been performed. **b** C-arm CT PBVI via the proper hepatic artery acquired following TACE showing an apparent complete response of the lesion to treatment. There is high density contrast in the lesion but no evidence of residual tumor perfusion. **c** Follow-up MDCT showing residual enhancing tumor which was subsequently shown to be supplied by the right inferior phrenic artery

The same angiography system was used in all procedures for C-arm CBCT acquisition (Artis Q, Siemens Healthcare AG, Forchheim, Germany); PBVI was performed before and after TACE. Post-processing was performed using Siemens Syngo DynaPBV Body (Siemens Healthcare AG, Forchheim, Germany). Each PBVI study involved two C-arm rotations. A catheter or microcatheter was positioned in the proper hepatic artery beyond the gastroduodenal artery origin. The first 5-s C-arm rotation was performed prior to contrast injection (mask run). Contrast injection was initiated immediately following this first rotation. After a 7-s delay as the C-arm returned to its starting position, a second 5-s CBCT was performed (fill run). Twelve milliliters of contrast agent (Omnipaque 350 mg I/mL, General Electric Healthcare, Oslo, Norway) was added to 24 mL of 0.9% saline solution, for a total volume of 36 mL. The contrast injection rate was 3 mL/s. The injection lasted 12 s. A total 17-s breath-hold was required. A non-rigid registration algorithm was used to correct for motion between the two CBCTs. Following segmentation of bone and air, the mask run was subtracted from the fill run allowing the calculation and color map depiction of blood volume values. Examples of the resulting images are shown in Figs. 1, 2 and 3. Conventional C-arm CBCT images were also reconstructed from the pre-TACE fill run to allow standard vessel mapping and procedure planning.

### MDCT/MRI protocol

Follow-up imaging was performed with multiphase MDCT or MRI. MDCT was performed using a 64-slice CT scanner (Siemens Somatom Sensation 64, Forchheim, Germany), with non-contrast, arterial, portal venous and 5-min delayed phase images. The contrast used was Omnipaque 350 mg I/mL (General Electric Healthcare, Oslo, Norway) at a dose of 150 mL MRI was performed on a 1.5-T scanner (Siemens Magnetom Avanto, Forchheim, Germany) and sequences including at least T1-weighted fat-suppressed non-contrast, arterial, portal venous and 5-min delayed phases. The gadolinium-based contrast agent used was gadoterate meglumine (Dotarem, Guerbet, Roissy, France) at a dose of 20 mL. In 17 cases, follow-up was performed with MDCT and in one case follow-up with MRI. The choice of follow-up imaging modality was dictated by the pre-procedure imaging modality in order to facilitate accurate comparison. Follow-up imaging was performed between 9 and 104 days (mean 60 days).

### Image analysis

All images were reviewed retrospectively by two interventional radiologists with 14-year (J.McC.) and 10-year (E.R.R.) experience in DEB-TACE by consensus. Analysis was lesion-based. Only lesions within the treated segment or segments were evaluated. In each case, a visual qualitative inspection was made of the post-TACE PBVI and an assessment was made for the presence or absence of residual tumor perfusion that would necessitate repeat TACE. Residual tumor perfusion requiring repeat TACE was recorded as either present or absent. The follow-up MDCT or MRI was analyzed in an identical manner, again for the presence or absence of residual enhancing tumor necessitating repeat TACE. As in the case of PBVI, residual lesion enhancement was recorded as present or absent.

### Statistical analysis

Sensitivity and specificity of post-TACE CBCT PBVI were calculated using follow-up MDCT/MRI as a reference standard. Point estimates were obtained along with their 95% confidence intervals.

## Results

In 9 of 18 cases, post-TACE PBVI was concordant with the follow-up cross-sectional imaging in demonstrating that no further treatment was required to the treated area (9 true negatives). In 6 of 18 cases, the post-TACE PBVI was concordant with the follow-up cross-sectional imaging in demonstrating that further treatment was required (6 true positives). In 3 of 18 cases, re-treatment was required based on residual tumor present on follow-up imaging that was not apparent on PBVI (3 false negatives). There were no false positives. The sensitivity of PBVI for detecting disease requiring repeat TACE was 67% (95% confidence interval 30–93%). The specificity of PBVI was 100% (95% confidence interval 66–100%).

## Discussion

Currently, the response to TACE is assessed on follow-up cross-sectional imaging (MDCT/MRI), with the disappearance of tumor enhancement representing a complete response by mRECIST criteria [10]. The clinical significance of the mRECIST criteria has been investigated and a treatment response has been shown to be associated with improved survival [11]. Following DEB-TACE, MDCT and MRI are similarly effective for the detection of residual viable tumor [12].

Perfusion imaging in HCC has shown promise but is not currently widely employed. Studies have found MDCT perfusion evaluation to be effective for detecting residual tumor following TACE for HCC and it is a viable modality for the assessment of treatment response [13, 14]. C-arm CBCT PBVI relies on the principle that the volume of blood in the arteries of a HCC reaches a steady state during prolonged contrast injection allowing the perfusion parameter of blood volume to be measured.

C-arm CBCT PBVI has previously been investigated in the setting of acute stroke [15], and there is interest in its applicability to HCC treatment. C-arm CBCT blood volume correlates well with MDCT perfusion blood volume in the liver [16, 17], and this approach has been shown to be reliable for the detection of HCC when compared with pre-procedure MDCT or MRI [18]. The same study also found it to be useful for a precise HCC localization and identification of their feeding vessels. In 2016, a study by Syha et al. [8] found that C-arm CBCT PBVI maps are useful in the post-treatment assessment of DEB-TACE in HCC and that residual tumor perfusion on PBVI maps can be used to predict mid-term tumor response [8]. Our study suggests that the use of C-arm CBCT PBVI immediately after TACE appears to have a high specificity for residual disease requiring repeat treatment, possibly allowing repeat TACE to be scheduled without the need for an intervening MDCT or MRI. This may have important implications for healthcare resource utilization, particularly in a resource-limited setting.

It is noteworthy that PBVI appears to have high specificity but limited sensitivity for detecting residual HCC requiring treatment. In our small sample, this low sensitivity is driven by the presence of three false negatives, without residual disease on PBVI but with residual viable tumor on follow-up MDCT/MRI (Fig. 3). There are two potential explanations for this. The first is the variable time to imaging follow-up after TACE, ranging from 9 to 104 days, with a mean of 60 days. In cases where follow-up imaging was delayed, tumor re-growth may have occurred leading to an apparent PBVI false negative. The second explanation comes from what may be the main limitation of CBCT PBVI, namely that only tumor lesions receiving supply from the proper hepatic artery (or whichever vessel contains the diagnostic catheter or microcatheter) will be detected. Lesions receiving extra-hepatic (or variant anatomical) arterial supply will not be detected on PBVI but will be apparent on MDCT/MRI. Therefore, a negative PBVI should be viewed with caution since the presence of residual disease is not excluded, although for the most part this will be predictable. As in the case of interval tumor re-growth, the potential for extra-hepatic arterial supply decreases the PBVI sensitivity but does not affect the specificity, *i.e.*, its ability to detect disease requiring further treatment.

Our study has a number of limitations. Firstly, it suffers from a small sample size (only 18 cases). This was a retrospective study with 43 TACE procedures with pre-treatment PBVI performed in the study period. However, only a subset of these also had post-TACE PBVI and follow-up imaging prior to further TACE. It was at the operator’s discretion whether to perform post-TACE PBVI, and this was not done in many cases, for example to due to an already long procedure time or elevated radiation dose. This may conceivably have introduced a selection bias. Next, there was heterogeneity in the DEB-TACE treatment protocol, and the exact TACE procedure parameters including catheter selection was at the discretion of the operator, although super-selective DEB-TACE was performed in all cases. Also, there was a wide range of time intervals for the follow-up MDCT or MRI. Our standard protocol includes cross-sectional imaging follow-up at 8–12 weeks. In some cases, imaging was performed earlier because of clinical concerns due to pain or fevers. This led to the wide range of intervals (9–104 days, mean 60 days).

In conclusion, the use of C-arm CBCT PBVI appears to be feasible for the detection of residual viable tumor within a treated lesion at the end of DEB-TACE, and this may allow a repeat TACE to be planned without the need to perform intervening cross-sectional imaging. Caution is required in an apparent complete response on C-arm CT PBVI due to the potential for false negatives. An apparent complete response at PBVI should be confirmed with MDCT or MRI.

## Abbreviations

BCLC: Barcelona clinic liver cancer staging system
CBCT: Cone-beam computed tomography
CT: Computed tomography
DEB-TACE: Drug-eluting bead transarterial chemoembolization
DSA: Digital subtraction angiography
HCC: Hepatocellular carcinoma
MDCT: Multidetector computed tomography
MRI: Magnetic resonance imaging
PBV: Parenchymal blood volume
PBVI: Parenchymal blood volume imaging
TACE: Transarterial chemoembolization

## References

[1] Gomaa AI, Khan SA, Toledano MB, Waked I, Taylor-Robinson SD (2008) Hepatocellular carcinoma: epidemiology, risk factors and pathogenesis. World J Gastroenterol 14:4300. 10.3748/wjg.14.4300 PMC2731180 18666317

[2] Llovet JM, Real MI, Montaña X et al (2002) Arterial embolisation or chemoembolisation versus symptomatic treatment in patients with unresectable hepatocellular carcinoma: a randomised controlled trial. Lancet 359:1734–1739. 10.1016/S0140-6736(02)08649-X 12049862

[3] Llovet JM, Bruix J (2003) Systematic review of randomized trials for unresectable hepatocellular carcinoma: chemoembolization improves survival. Hepatology 37:429–442. 10.1053/jhep.2003.50047 12540794

[4] Forner A, Gilabert M, Bruix J, Raoul JL (2014) Treatment of intermediate-stage hepatocellular carcinoma. Nat Rev Clin Oncol 11:525–535. 10.1038/nrclinonc.2014.122 25091611

[5] Tognolini A, Louie JD, Hwang GL, Hofmann LV, Sze DY, Kothary N (2010) Utility of C-arm CT in patients with hepatocellular carcinoma undergoing transhepatic arterial chemoembolization. J Vasc Interv Radiol 21:339–347. 10.1016/j.jvir.2009.11.007 20133156

[6] Iwazawa J, Ohue S, Hashimoto N, Muramoto O, Mitani T (2012) Survival after C-arm CT-assisted chemoembolization of unresectable hepatocellular carcinoma. Eur J Radiol 81:3985–3992. 10.1016/j.ejrad.2012.08.012 22959287

[7] Loffroy R, Lin M, Yenokyan G et al (2013) Intraprocedural C-arm dual-phase cone-beam CT: can it be used to predict short-term response to TACE with drug-eluting beads in patients with hepatocellular carcinoma. Radiology 266:636–648. 10.1148/radiol.12112316 PMC3558876 23143027

[8] Syha R, Grözinger G, Grosse U et al (2016) Parenchymal blood volume assessed by C-arm-based computed tomography in immediate posttreatment evaluation of drug-eluting bead transarterial chemoembolization in hepatocellular carcinoma. Invest Radiol 51:121–126. 10.1097/RLI.0000000000000215 26488373

[9] Vogl TJ, Schaefer P, Lehnert T et al (2016) Intraprocedural blood volume measurement using C-arm CT as a predictor for treatment response of malignant liver tumours undergoing repetitive transarterial chemoembolization (TACE). Eur Radiol 26:755–763. 10.1007/s00330-015-3869-y 26123407

[10] Lencioni R, Llovet JM (2010) Modified RECIST (mRECIST) assessment for hepatocellular carcinoma. Semin Liver Dis 30:52–60. 10.1055/s-0030-1247132 PMC12268942 20175033

[11] Vincenzi B, Di Maio M, Silletta M et al (2015) Prognostic relevance of objective response according to EASL criteria and mRECIST criteria in hepatocellular carcinoma patients treated with loco-regional therapies: a literature-based meta-analysis. PLoS One 10:e0133488. 10.1371/journal.pone.0133488 PMC4521926 26230853

[12] Kloeckner R, Otto G, Biesterfeld S, Oberholzer K, Dueber C, Pitton MB (2010) MDCT versus MRI assessment of tumor response after transarterial chemoembolization for the treatment of hepatocellular carcinoma. Cardiovasc Intervent Radiol 33:532–540. 10.1007/s00270-009-9728-y 19847482

[13] Ippolito D, Bonaffini PA, Ratti L et al (2010) Hepatocellular carcinoma treated with transarterial chemoembolization: dynamic perfusion-CT in the assessment of residual tumor. World J Gastroenterol 16:5993–6000. 10.3748/wjg.v16.i47.5993 PMC3007114 21157976

[14] Ippolito D, Fior D, Bonaffini PA et al (2014) Quantitative evaluation of CT-perfusion map as indicator of tumor response to transarterial chemoembolization and radiofrequency ablation in HCC patients. Eur J Radiol 83:1665–1671. 10.1016/j.ejrad.2014.05.040 24962900

[15] Fiorella D, Turk A, Chaudry I et al (2014) A prospective, multicenter pilot study investigating the utility of flat detector derived parenchymal blood volume maps to estimate cerebral blood volume in stroke patients. J Neurointerv Surg 6:451–456. 10.1136/neurintsurg-2013-010840 PMC4112493 23943817

[16] Peynircioğlu B, Hızal M, Çil B et al (2015) Quantitative liver tumor blood volume measurements by a C-arm CT post-processing software before and after hepatic arterial embolization therapy: comparison with MDCT perfusion. Diagn Interv Radiol 21:71–77. 10.5152/dir.2014.13290 PMC4463359 25538037

[17] Zhuang ZG, Zhang XB, Han JF et al (2014) Hepatic blood volume imaging with the use of flat-detector CT perfusion in the angiography suite: comparison with results of conventional multislice CT perfusion. J Vasc Interv Radiol 25:739–746. 10.1016/j.jvir.2014.01.021 24745904

[18] Syha R, Grözinger G, Grosse U et al (2015) C-arm computed tomography parenchymal blood volume measurement in evaluation of hepatocellular carcinoma before transarterial chemoembolization with drug eluting beads. Cancer Imaging 15:22. 10.1186/s40644-015-0057-x PMC4696182 26715200

